# Competing with a pandemic: Trends in research design in a time of Covid-19

**DOI:** 10.1371/journal.pone.0238831

**Published:** 2020-09-10

**Authors:** Shelly X. Bian, Eugene Lin

**Affiliations:** 1 Department of Radiation Oncology, University of Southern California, Los Angeles, California, United States of America; 2 Division of Nephrology and Hypertension, Department of Medicine, University of Southern California, Los Angeles, California, United States of America; London School of Economics and Political Science, UNITED KINGDOM

## Abstract

**Introduction:**

During the Covid-19 pandemic, major journals have published a significant number of Covid-19 related articles in a short period of time. While this is necessary to combat the worldwide pandemic, it may have trade-offs with respect to publishing research from other disciplines.

**Objectives:**

To assess differences in published research design before and after the Covid-19 pandemic.

**Methods:**

We performed a cross-sectional review of all 322 full-length research studies published between October 1, 2019 and April 30, 2020 in three major medical journals. We compared the number of randomized controlled trials (RCTs) and studies with a control group before and after January 31, 2020, when Covid-19 began garnering international attention.

**Results:**

The number of full-length research studies per issue was not statistically different before and after the Covid-19 pandemic (from 3.7 to 3.5 per issue, p = 0.17). Compared to before January 31, 2020, 0.7 fewer non-Covid-19 studies per issue were published versus after January 31, 2020 (p<0.001), a change that was offset by Covid-19 studies. Among non-Covid-19 studies, 0.9 fewer studies with a control group per issue were published after January 31, 2020, with RCTs contributing to nearly all the decline (p<0.001, p = 0.001, respectively). In the same timeframe, non-Covid-19 studies without a control group and non-Covid-19 studies without randomization experienced relatively small changes that did not meet our threshold for statistical significance (increases of 0.1 and 0.1 per issue, p = 0.80, p = 0.88, respectively).

**Limitations:**

Using a simple heuristic for assessing research design and lack of generalizability to the general medical literature.

**Conclusions:**

In summary, the increase in Covid-19 studies coincided with a decrease of mostly non-Covid-19 RCTs.

## Introduction

The Covid-19 pandemic has severely disrupted medical research, from shutting down laboratory facilities, to delaying clinical trials, to halting funding [1, 2]. Simultaneously, the volume of Covid-19 research has proliferated as experts from all disciplines seek to combat the new threat [3, 4]. In the months following the onset of Covid-19, there has been an unprecedented increase in the number of registered clinical trials, preprints, and publications related to Covid-19 [5].

As of May, 2020, ClinicalTrials.gov listed over 1000 Covid-19 studies. Although some will advance our clinical knowledge, many are small, poorly designed, redundant, and unlikely to prove clinically useful [6]. Meanwhile the peer review process for Covid-19 studies has accelerated substantially. An analysis of 14 medical journals found a 50% reduction in average turnaround time from submission to publication for Covid-19 related research. This study, however, did not include several of the most influential journals including *The Journal of the American Medical Association*, *The Lancet*, and *The New England Journal of Medicine* [7]. The proliferation of Covid-19 literature has permeated into non-peer reviewed preprint websites, such as BioRxiv and MedRxiv. As of May 7, MedRxiv and BioRxiv had close to 2800 Covid-19 related articles combined. This has led to several editorials expressing concern over the quality of Covid-19 related research [8, 9].

Many people look to most influential medical journals as having the highest standards for publication. The shifting of our research priorities is appropriate and necessary to address the pandemic. However, this diversion may also have unintended consequences on research in other disciplines. To evaluate whether the Covid-19 pandemic had negative effects on published research in other disciplines, we studied characteristics of Covid-19 and non-Covid-19 related research in three of the most cited medical journals: *The Journal of the American Medical Association*, *The Lancet*, and *The New England Journal of Medicine*. Although prior studies have evaluated the proliferation, quality, and design of *Covid-19 related* publications, none to our knowledge have addressed the pandemic’s effect on the study design of non-Covid-19 medical research. In this cross-sectional study, we investigated whether the design of published non-Covid-19 research changed in three of the highest impact medical journals aimed at a predominantly clinical audience after the onset of the Covid-19 pandemic.

## Methods

### Identifying research studies

We reviewed all full-length research studies published between October 1, 2019 and April 30, 2020 in three of the highest impact medical journals, *The New England Journal of Medicine (NEJM)*, the *Journal of the American Medical Association (JAMA)*, and *The Lancet*. These included “Original Articles” from *NEJM* (including “Brief Reports” and “Special Reports”), “Original Research” from *JAMA* (including “Preliminary Communications” and “Special Communications”), and “Research Articles” from *The Lancet*.

**Fig 1:**
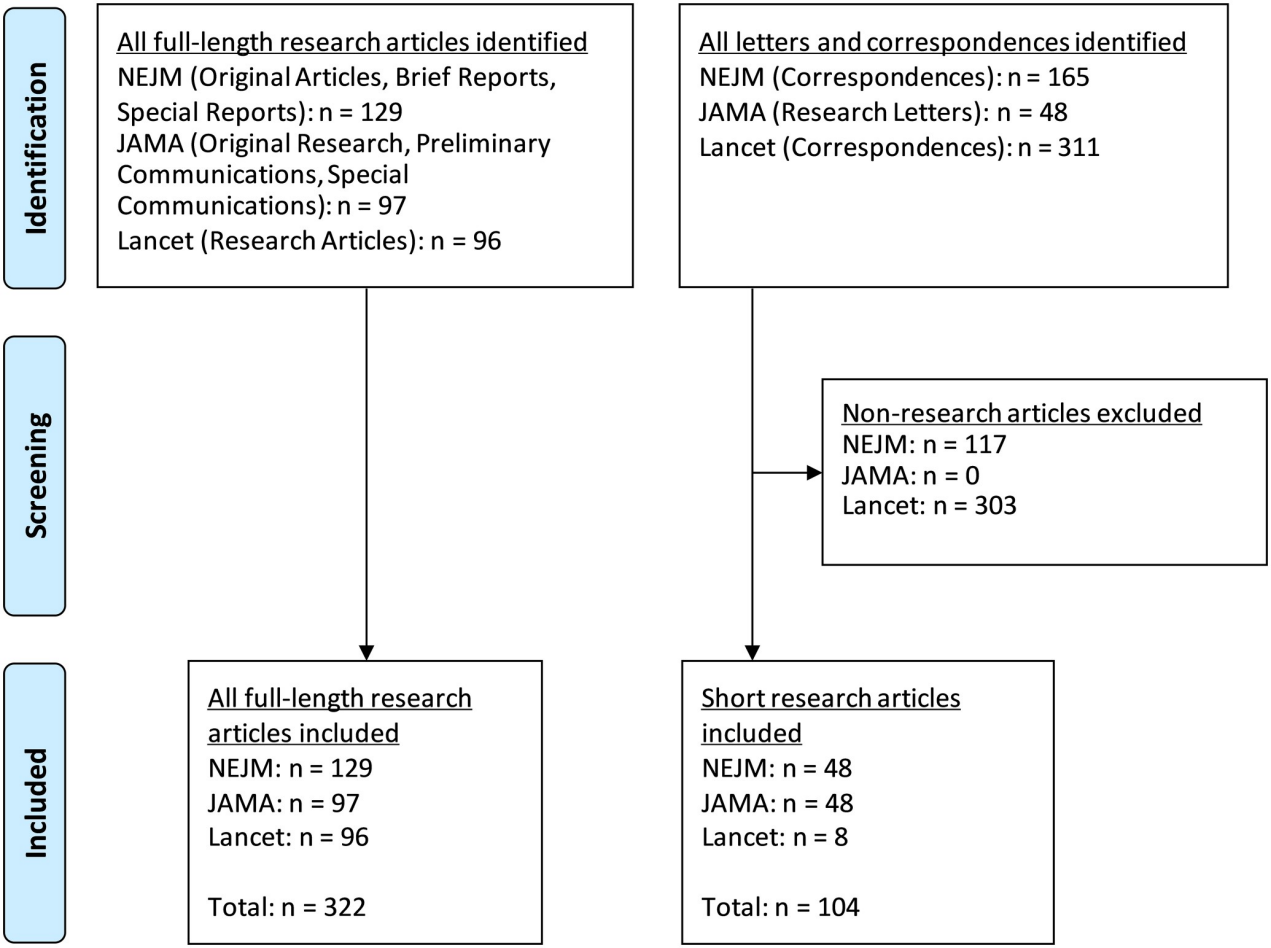
PRISMA flow diagram.

Many Covid-19 studies are case series published as short research studies and letters to the editor. In a sensitivity analysis, we additionally included short research studies published in letter format: “Correspondences” from *NEJM* and *The Lancet* and “Research Letters” from *JAMA*. The Correspondence sections from the *NEJM* and *The Lancet* have a broad range and include letters addressing previous articles, primary research, and opinions. From these, we manually identified all letters with a primary data collection effort (including case series and translational research) or secondary analysis of already collected data. We included all of *JAMA’s* Research Letters. We excluded sections dedicated for case reports (e.g., the “Case Records from the Massachusetts General Hospital in *The New England Journal of Medicine*” or “Brief Reports” from *JAMA*) but included case reports or case series published as short research studies or letters to the editor. Our publication selection process is outlined in a Prisma flow diagram in Fig 1.

We assigned each study an organ system (S1 Table in S1 Appendix) and determined whether it was (i) Covid-19 related, (ii) a study with a control group, and (iii) a randomized controlled trial (RCT). We defined Covid-19-related research as having any Covid-19 related words in the title of the article including “Covid-19”, “novel coronavirus”, “2019-nCoV”, “Coronavirus disease 2019”, or SARS-CoV-2”. We defined a control group as whether the investigators performed a hypothesis-driven analysis, comparing two or more groups. Studies describing trends over time were considered uncontrolled unless the investigators tested a specific hypothesis, such as analyzing the putative effect of a policy intervention. Both authors reviewed all studies, adjudicating conflicts by consensus (see S1 Appendix for additional formal designations).

### Statistical analyses

We compared study characteristics before and after the end of January 2020, when Covid-19 began garnering international attention. We defined the pre-Covid-19 era as January 31, 2020 and prior, and the post-Covid-19 era as February 1, 2020 and after. We chose this cutoff because the last week of January, 2020 marked the public announcement of the first cases of Covid-19 outside of China. Simultaneously, the World Health Organization (WHO) began warning other countries of the virus’ global spread and the importance of formulating strategic plans for virus containment around this time. Furthermore, the first Covid-19 publication in the three medical journals of interest was published on February 15, 2020 in *The Lancet*.

We first plotted the number of published Original Research studies, studies that were Covid-19 related, studies with and without a control group, and studies that were RCTs over time. We performed this descriptive analysis by assigning each issue to a given week, anchoring to the Monday of that week. We computed the average number of studies per week before and after the end of January 2020 to visually demonstrate changes in before and after Covid-19.

Subsequently, we assessed per issue differences in study type before and after the end of January 2020. For each of the three journals examined, issues are published weekly with the rare exception of an omitted week. We used the Mann-Whitney-Wilcoxan test to assess whether the average number of Covid-19 related studies, studies with and without a control group, and studies that were RCTs per issue was different before and after January 31, 2020. We performed analyses using the *number* of studies per issue instead of the proportion of studies per issue because counts reflect the zero-sum nature of publications and provide meaningful information on the volume of studies published. For instance, a large decrease in the total number of published studies might not change the proportion of published RCTs but would likely decrease the number of published RCTs. Because the total number of published studies per issue did not change substantially over time, this distinction was unlikely material to our results.

Our primary focus was on all full-length research studies and on the non-Covid-19 related subgroup of studies. In a sensitivity analyses, we expanded our sample to all research studies including those in letter format.

To assess changes in publication by organ system, we aggregated the total number of full-length research articles into pre-Covid-19 and post-Covid-19 cohorts. We did this because of the scarcity of some organ systems at an issue level. Because the pre-Covid-19 study period had more months, we plotted the percent of published full-length research studies before and after the onset of Covid-19. For each organ system, we compared the significance of the change in proportion before and after Covid-19 using Fisher’s exact test.

We acknowledge that we did not pre-register our study with a predetermined statistical protocol. Additionally, we performed a large number of statistical tests. Therefore, we performed a conservative Bonferroni correction [10] to adjust the threshold for rejecting the null hypothesis. Because we performed 21 tests in our primary analysis, we required a p-value of 0.0024 when determining whether a difference was statistically significant.

## Results

We identified 322 total full-length research studies, 188 before and 134 after the end of January, 2020. The distribution of full-length research articles by journal for each month is detailed in Table 1.

**Table 1:** Distribution of research article type by journal.

Month	NEJM			JAMA			Lancet		
	Full-length article	Research letter	Total	Full-length article	Research letter	Total	Full-length article	Research letter	Total
October	20	8	28	14	5	19	14	0	14
November	17	4	21	15	7	22	17	2	19
December	17	5	22	13	8	21	12	1	13
January	23	8	31	14	8	22	12	1	13
February	17	4	21	14	6	20	15	0	15
March	15	9	24	14	5	19	12	3	15
April	20	10	30	13	9	22	14	1	15
Total	129	48	177	97	48	145	96	8	104

**Abbreviations**: NEJM = The New England Journal of Medicine, JAMA = Journal of the American Medical Association.

**Fig 2:**
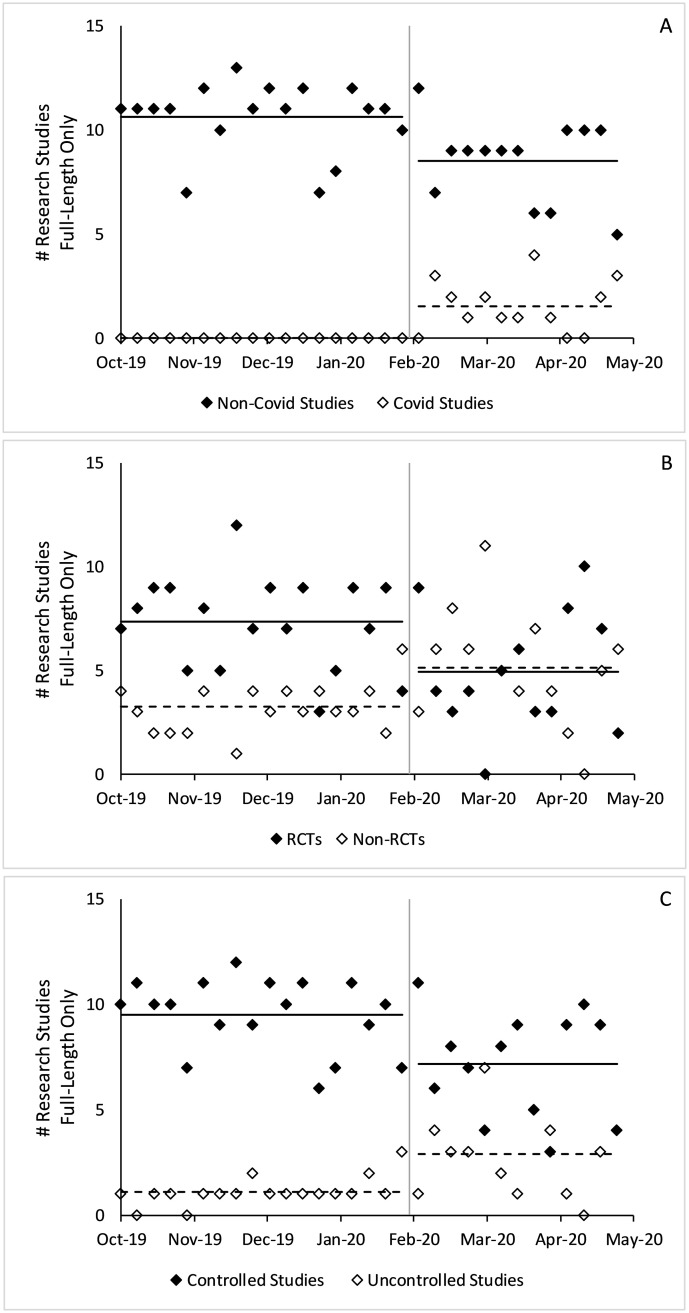
Changes in full-length research studies published before and after Covid-19. Figure includes all full-length research studies in counts per week. A) Covid-19 versus non-Covid-19 studies, B) controlled versus uncontrolled studies, C) RCTs versus non-RCTs. P-values estimated using a two-sample t-test. The gray vertical lines denote January 31, 2020, the boundary between the pre- and post-Covid-19 eras. Solid horizontal lines denote average values of the solid dots and dashed horizontal lines denote average values of the hollow dots.

The total number of full-length research studies did not statistically differ before and after Covid-19 (from 3.7 to 3.5 per issue, p = 0.17). After January 2020, non-Covid-19 full-length studies decreased by 0.7 per issue on average (p<0.001), which was offset by Covid-19 full-length studies. The total number of full-length studies with a control group decreased by 0.8 per issue on average (p<0.001) and the number of RCTs decreased by 0.9 per issue (p = 0.001). Fig 2 shows changes in full-length articles by week, which usually includes 1 issue from each journal.

**Fig 3:**
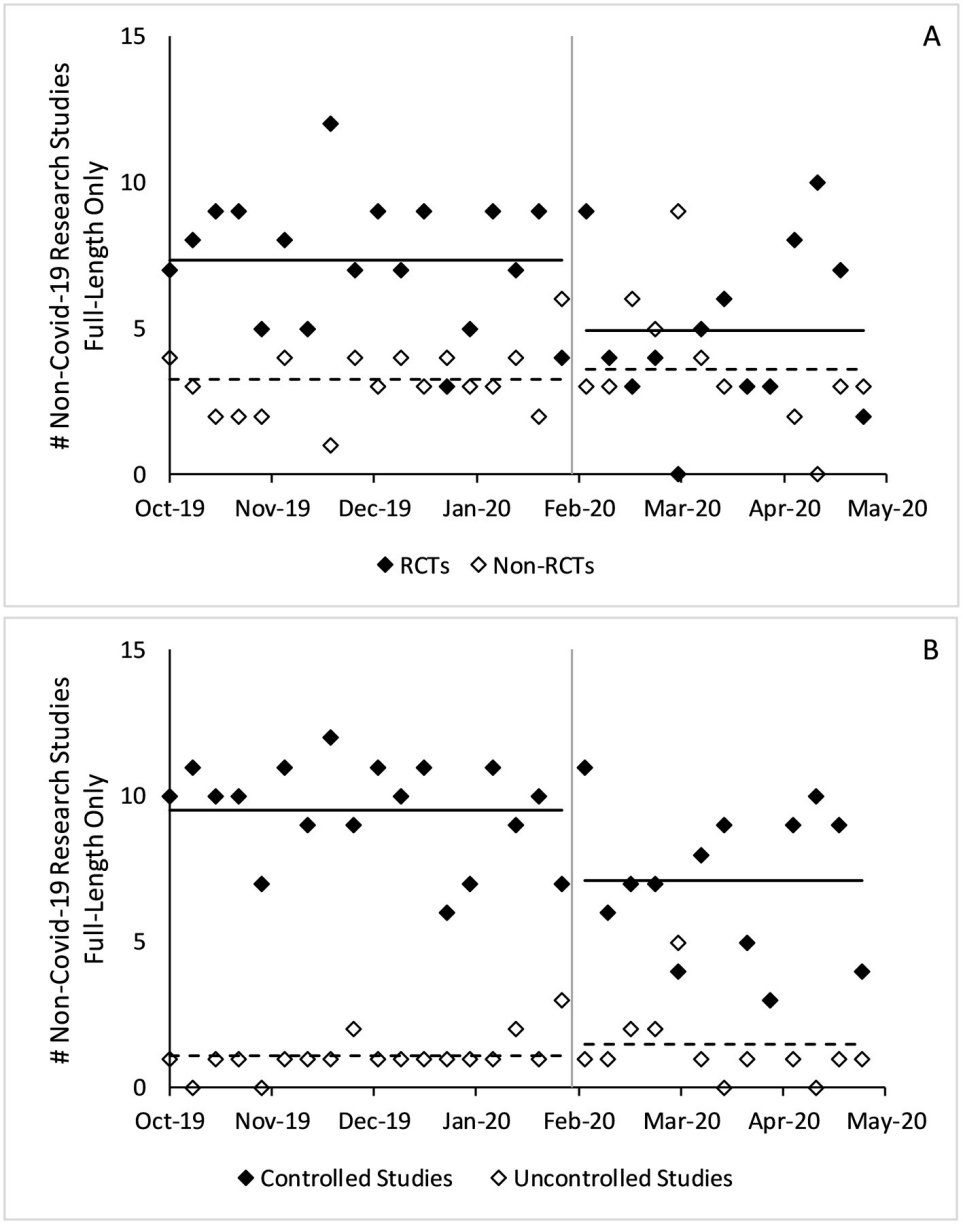
Changes in full-length non-Covid-19 research studies published before and after Covid-19. Figure includes all non-Covid-19 full-length research studies in counts per week. A) controlled versus uncontrolled studies B) RCTs versus non-RCTs. P-values estimated using a two-sample t-test. The gray vertical lines denote January 31, 2020, the boundary between the pre- and post-Covid-19 eras. Solid horizontal lines denote average values of the solid dots and dashed horizontal lines denote average values of the hollow dots.

Among non-Covid-19 full-length studies, the number of studies with a control group decreased by 0.9 per issue on average with RCTs contributing to nearly all the decline (p<0.001, p = 0.001, respectively). In the same timeframe, non-Covid-19 full-length studies without a control group and non-Covid-19 full-length studies without randomization did not statistically differ (p = 0.80, 0.88, respectively). Fig 3 shows weekly changes in full-length non-Covid-19 articles.

**Fig 4:**
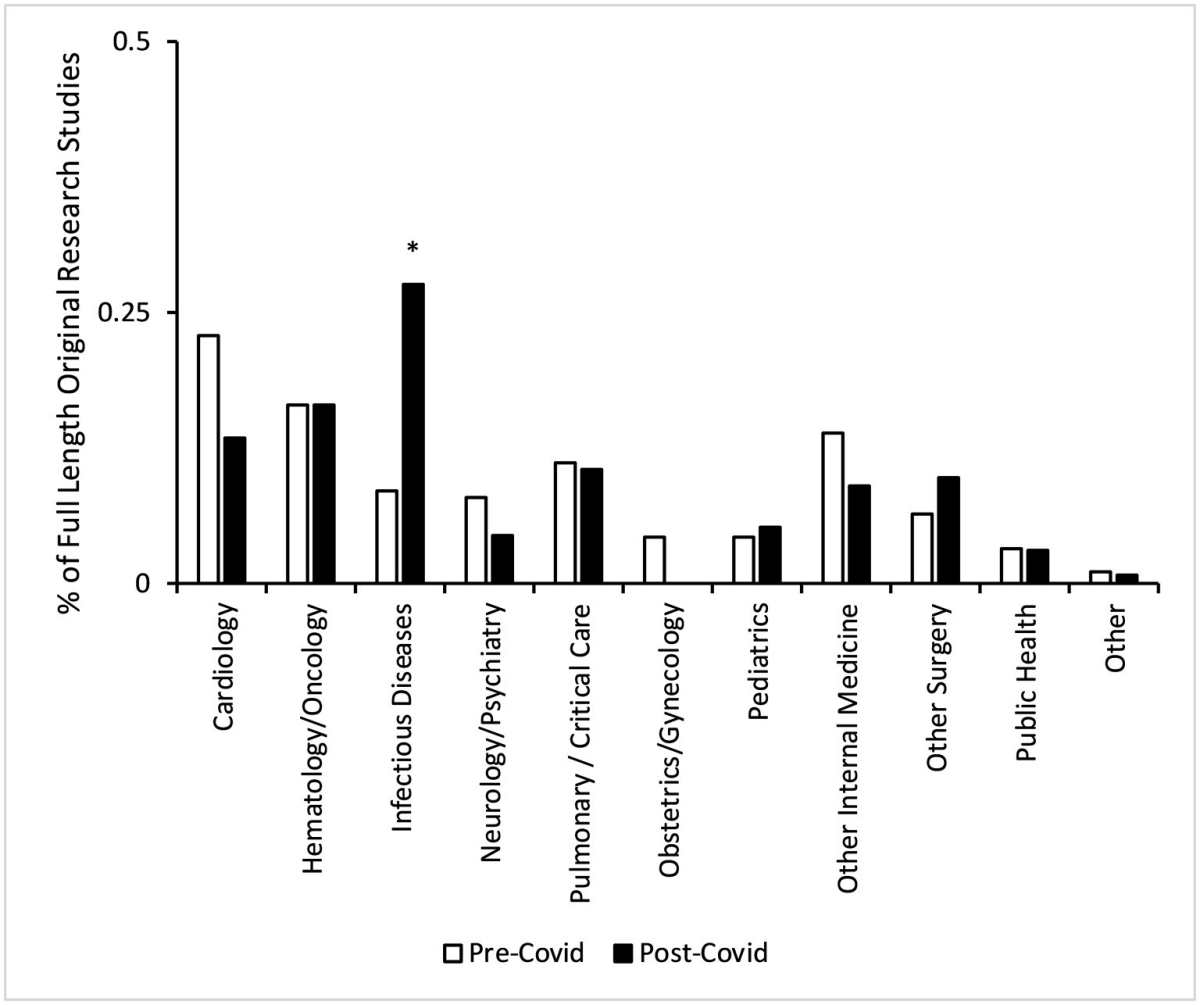
Distribution of full-length research studies pre- and post-Covid-19 by specialty. P-values were estimated using Fisher’s Exact Test. (an asterisk, *, denotes p < 0.0025). January 31, 2020 is the boundary between the pre- and post-Covid-19 eras.

Cardiology and obstetrics/gynecology had the largest declines in full-length published studies, from 23% to 13% (9% decline, p = 0.04) and from 4% to 0% (4% decline, p = 0.02, respectively. Meanwhile infectious diseases had the largest increase, from 9% to 28% (19% increase, p<0.001) (Fig 4, Table 2).

**Table 2:** Change in full-length research articles pre- and post-Covid-19 by specialty.

Organ System	Pre-Covid Number(%)*	Post-Covid Number (%)	Difference**	p-value***
Cardiology	43 (22.9%)	18 (13.4%)	–9.4%	0.04
Hematology/Oncology	31 (16.5%)	22 (16.4%)	–0.1%	1.00
Infectious Diseases	16 (8.5%)	37 (27.6%)	19.1%	**<0.001**
Neurology/Psychiatry	15 (8.0%)	6 (4.5%)	–3.5%	0.26
Pulmonary / Critical Care	21 (11.2%)	14 (10.4%)	–0.7%	1.00
Obstetrics/Gynecology	8 (4.3%)	0 (0%)	–4.3%	0.02
Pediatrics	8 (4.3%)	7 (5.2%)	1.0%	0.79
Other Internal Medicine	26 (13.8%)	12 (9.0%)	–4.9%	0.22
Other Surgery	12 (6.4%)	13 (9.7%)	3.3%	0.30
Public Health	6 (3.2%)	4 (3.0%)	–0.2%	1.00
Other	2 (1.1%)	1 (0.7%)	–0.3%	1.00

* % denotes percentage of all full-length articles.

** The absolute difference when going from the pre-Covid to post-Covid periods.

***p-values were obtained using Fisher’s Exact Test. A p-value < 0.0025 was considered statistically significant.

In a sensitivity analysis, we included an additional 104 short research studies (57 pre- and 47 post-Covid-19) typically published in Letter format. The sensitivity analysis included a total of 426 research studies, 425 pre- and 181 post-Covid-19 and was not materially different from our primary analysis.

**Fig 5:**
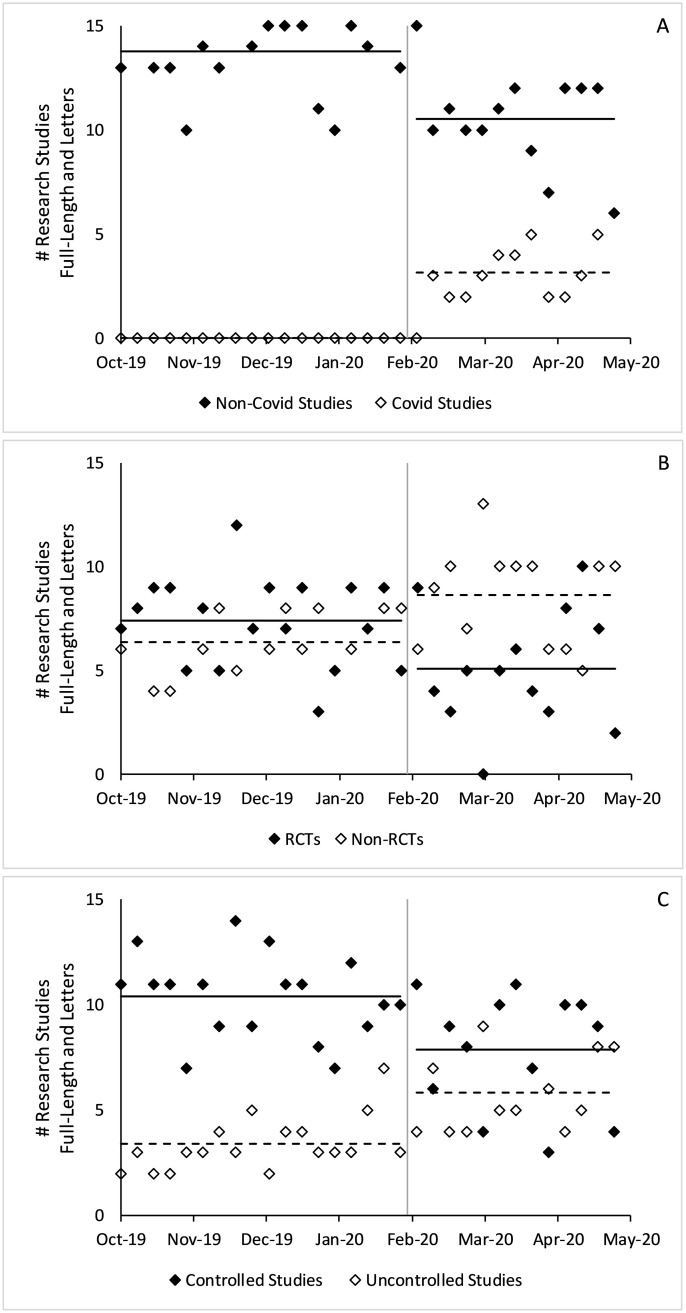
Changes in all research studies published before and after Covid-19. Figure includes full-length research studies as well as research studies in Letter form in counts per week. A) Covid-19 versus non-Covid-19 studies, B) controlled versus uncontrolled studies, C) RCTs versus non-RCTs. P-values estimated using a two-sample t-test. The gray vertical lines denote January 31, 2020, the boundary between the pre- and post-Covid-19 eras. Solid horizontal lines denote average values of the solid dots and dashed horizontal lines denote average values of the hollow dots.

The total number of studies overall did not statistically differ before and after Covid-19 (from 4.9 to 4.8 per issue, p = 0.95). After January 31, 2020, non-Covid-19 studies decreased by 1.2 per issue on average (p<0.001), which was offset by Covid-19 studies. We observed a decrease in number of studies pre- and post-Covid-19 with a control group of 0.9 per issue on average (p = 0.001), which was almost entirely explained by a decrease in RCTs (p = 0.002). Fig 5 shows weekly changes in all articles.

**Fig 6:**
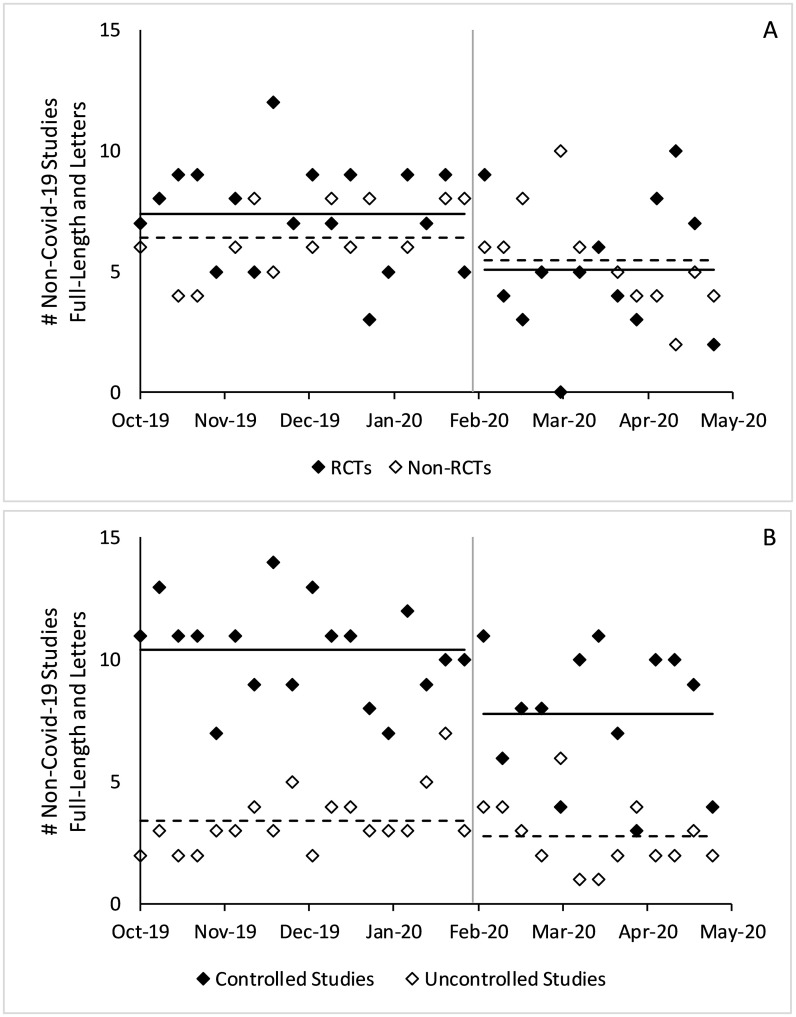
Changes in all non-Covid-19 research studies published before and after Covid-19. Figure includes all non-Covid-19 full-length research studies as well as research studies in Letter form. A) controlled versus uncontrolled studies B) RCTs versus non-RCTs. P-values estimated using a two-sample t-test.

Among non-Covid-19 studies, the number of studies with a control group decreased by 0.9 per issue on average with RCTs contributing to nearly all the decline (p<0.001, p = 0.002, respectively). In the same timeframe, non-Covid-19 studies without a control group and non-Covid-19 studies without randomization did not statistically differ (p = 0.27, 0.26, respectively). Fig 6 shows weekly changes in all non-Covid-19 articles.

In contrast, none of the Covid-19 studies (20 full-length and 21 in letter format) were RCTs, and only one had a control group. Table 3 shows changes in articles per issue in all articles as well as full-length articles before and after January 31, 2020.

**Table 3:** Changes in articles per issue before and after January 31, 2020, the boundary between the defined pre- and post-Covid eras.

	Average count per issue			
	Pre-Covid	Post-Covid	Absolute Change	p-value *
**All Full-Length Articles**	3.7	3.5	–0.2	0.17
RCTs	2.6	1.7	–0.9	**0.001**
non-RCTs	1.2	1.8	0.7	0.007
Controlled Studies	3.4	2.5	–0.8	**< 0.001**
Uncontrolled Studies	0.4	1.0	0.6	0.006
** Covid Full-Length Articles**	–	0.5	–	–
RCTs	–	0.0	–	–
non-RCTs	–	0.5	–	–
Controlled Studies	–	0.0	–	–
Uncontrolled Studies	–	0.5	–	–
** Non-Covid Full-Length Articles**	3.7	3.0	–0.7	**< 0.001**
RCTs	2.6	1.7	–0.9	**0.001**
non-RCTs	1.2	1.3	0.1	0.88
Controlled Studies	3.4	2.5	–0.9	**< 0.001**
Uncontrolled Studies	0.4	0.5	0.1	0.80
**All Articles**	4.9	4.8	–0.1	0.95
RCTs	2.6	1.8	–0.8	**0.002**
non-RCTs	2.3	3.0	0.8	0.004
Controlled Studies	3.7	2.8	–0.9	**0.001**
Uncontrolled Studies	1.2	2.1	0.9	**0.002**
** All Covid Articles**	–	1.1	–	–
RCTs	–	0.0	–	–
non-RCTs	–	1.1	–	–
Controlled Studies	–	0.0	–	–
Uncontrolled Studies	–	1.1	–	–
** All Non-Covid Articles**	4.9	3.7	–1.2	**< 0.001**
RCTs	2.6	1.8	–0.8	**0.002**
non-RCTs	2.3	1.9	–0.3	0.26
Controlled Studies	3.7	2.7	–0.9	**< 0.001**
Uncontrolled Studies	1.2	1.0	–0.2	0.27

* Comparisons performed using the Mann-Whitney-Wilcoxan test to compare the number studies falling in each category per issue before and after Covid-19. A p-value < 0.0025 was considered statistically significant.

Abbreviations: RCTs = randomized controlled trial.

## Discussion

We found that in three of the highest impact medical journals, the increase in Covid-19-related research studies coincided with a concomitant decrease in non-Covid-related RCTs. Unsurprisingly, most Covid-19 studies published in the first three months of the pandemic comprised case reports and case series. No Covid-19 related RCT’s were published from February 1, to April 30, 2020. We observed a decreasing trend in cardiology and obstetrics/gynecology studies post-Covid-19, though the decrease did not meet our threshold for statistical significance.

Prioritizing Covid-19 research is critical to combating the pandemic [1]. The medical community needs and expects the quick dissemination of Covid-19 research. However, hasty research may result in suboptimal study designs, though some have argued for the need to balance scientific rigor for speed [11]. Similarly, Kim et. al. recently argued in the *Annals of Internal Medicine* has stated that “Given the urgency of the situation, some limitations… may be acceptable, including the small sample size, use of an unvalidated surrogate end point, and lack of randomization or blinding” [8]. Still, researchers and publishers must use caution. In the last month alone, four high-profile Covid-19 articles have been retracted from the *Annals of Internal Medicine*, *The Lancet*, and *NEJM* due to inadequate scientific rigor [12–15]. We similarly observed a large increase in Covid-19 studies that did not have a control group, mostly case reports and series. As Covid-19 researchers have time to apply more rigorous methods to their studies, RCTs and studies with control groups will undoubtedly become more widespread. In the meantime, researchers and journal editors will need to balance the trade-off between accommodating the rapid dissemination of information with Covid-19 research that do not report a control group.

However, we were concerned to observe substitutions occurring at the expense of non-Covid-19 studies with a control group, with substitutions occurring almost entirely at the expense of RCTs. In comparison, we observed minimal changes among non-Covid-19 studies without a control group. If we project these changes over a course of 6 months (a conservative time frame for the Covid-19 pandemic), non-Covid-19 studies with a control group could decrease by 60 and non-Covid-19 RCTs by 70 in these three medical journals combined. Notably, in the field of obstetrics/gynecology, zero studies were published in the post-Covid-19 era despite constituting 4% of full-length publications prior to the outbreak. The decline in non-Covid-19 publications with a control group and especially RCTs likely has multiple reasons. Publication of already accepted studies may be postponed in lieu of emerging Covid-19 research. Journals in the JAMA Network have received 53% more submissions in the first quarter of 2020 than in the first quarter of 2019 [16]. The flood of submissions and the demand for Covid-19 related work presents publishers and editors with the dilemma of accepting fewer non-Covid-19 publications, quality notwithstanding.

Reviewers and editors might also perceive non-Covid-19 research as overall less relevant given the pandemic. The WHO, national disease organizations, and media have Covid-19 in the forefront of public health. Since January, 2020, COVID-19 papers have been downloaded more than 150 million times, according to the International Association of Scientific, Technical and Medical Publishers [17]. Major publications have a responsibility to assess the interest of their readership and adjust their publication profile accordingly. Finally, authors might delay submissions until after the pandemic subsides because of unavoidable disruptions in their research or a perception that non-Covid-19 research is viewed less favorably. Many laboratories and non-Covid-19 clinical trials have closed due to social distancing [18]. Additionally, resources and priorities may have shifted away from non-Covid-19 research to support Covid-19 studies [19].

Unfortunately, these rejections and delays contribute to a growing backlog of unpublished non-Covid-19 research, hindering future dissemination. The backlog will likely worsen as non-Covid-19 research resumes with long-term negative consequences to published research [9]. Disruptions in medical research attributable to Covid-19 could thus mirror public health experts’ concerns about delays in non-Covid-19 medical care [20].

Limitations of our study include using a simple heuristic for assessing study design (e.g., RCT versus not, controlled versus uncontrolled), not accounting for contemporaneous idiosyncratic events (e.g., vaping), and not observing submitted but unpublished work. Additionally, we did not produce a written protocol and preplanned analysis prior to data collection. We also limited our sample to three select high impact journals, and our results do not necessarily generalize to the entire medical literature. We were unable to estimate changes pre- and post-Covid-19 by organ system with precision, likely owing to small sample size. Finally, we used the 3 months preceding the start of Covid-19 to assess the pre-Covid-19 baseline for the three journals of interest. However this surrogate may not be a suitable counterfactual for these journals in the absence of Covid-19. Thus, our findings should be corroborated in a wider sample of journals and time periods.

Substitutions of non-Covid-19 research are inevitable. To alleviate delays in publication, editors could consider establishing dedicated channels for Covid-19 studies that do not compete with non-Covid-19 studies.

## Supporting information

S1 Appendix Additional details for “Competing with a Pandemic: Research Quality in a Time of Covid-19”. (DOCX)
