# Supplementary material for: Competing with a pandemic: Trends in research design in a time of Covid-19
Source: PLoS One. 2020 Sep 10;15(9):e0238831. doi: 10.1371/journal.pone.0238831 (PMC7482957; doi:10.1371/journal.pone.0238831)
Supplement: S1 Appendix — (DOCX) [file pone.0238831.s001.docx]

S1 Appendix: Additional Details for “Competing with a Pandemic: Research Quality in a Time of Covid-19”

After initial data collection, studies were classified by type (Brief Report-Original Research, Correspondence, Correspondence-Covid, Original Research, Preliminary Communication, Research Article, Research Letter, Special Communication, and Special Report). We categorized short articles (typically less than 700 words) as “Letters:” Correspondences, Correspondences-Covid, and Research Letters. We defined all other articles as full-length Research studies.

We next categorized studies into organ systems (**S2 Table**). Because of the large number of categories, we further consolidated organ systems that were similar and had low numbers of articles.

**S1 Table: Schematic for Categorizing Articles into Organ System**

| **Original Organ Classification** | **Organ Classification for Analysis** |
| --- | --- |
| Allergy/immunology | Other Internal Medicine |
| Anesthesia | Other |
| Cardiology | Cardiology |
| Critical care | Pulmonary / Critical Care |
| Dermatology | Other Internal Medicine |
| Endocrinology | Other Internal Medicine |
| Otolaryngology | Other Surgery |
| Epidemiology | Public Health |
| General | Other Internal Medicine |
| Gastroenterology | Other Internal Medicine |
| Gynecology | Obstetrics / Gynecology |
| Health Economics | Public Health |
| Hematology | Hematology / Oncology |
| Infectious Diseases | Infectious Diseases |
| Nephrology | Other Internal Medicine |
| Neurology | Neurology / Psychiatry |
| Obstetrics | Obstetrics / Gynecology |
| Oncology | Hematology / Oncology |
| Ophthalmology | Other Surgery |
| Orthopedics | Other Surgery |
| Pathology | Other |
| Pediatrics | Pediatrics |
| Physiology | Other |
| Psychiatry | Neurology / Psychiatry |
| Public Health | Public Health |
| Pulmonology | Pulmonary / Critical Care |
| Radiology | Other |
| Research | Other |
| Rheumatology | Other Internal Medicine |
| Surgery | Other Surgery |
| Trainee | Other |
| Transplant | Other |
